# Preclinical assessment of the VEGFR inhibitor axitinib as a therapeutic agent for epithelial ovarian cancer

**DOI:** 10.1038/s41598-020-61871-w

**Published:** 2020-03-17

**Authors:** E Sun Paik, Tae-Hyun Kim, Young Jae Cho, Jiyoon Ryu, Jung-Joo Choi, Yoo-Young Lee, Tae-Joong Kim, Chel-Hun Choi, Woo Young Kim, Jason K. Sa, Jin-Ku Lee, Byoung-Gie Kim, Duk-Soo Bae, Hee Dong Han, Hyung Jun Ahn, Jeong-Won Lee

**Affiliations:** 1 Department of Obstetrics and Gynecology, Kangbuk Samsung Hospital, Sungkyunkwan University School of Medicine, Seoul, Republic of Korea; 2 Department of Obstetrics and Gynecology, Konyang University Hospital, Daejon, Republic of Korea; 3 Department of Obstetrics and Gynecology, Samsung Medical Center, Sungkyunkwan University School of Medicine, Seoul, Republic of Korea; 4 Department of Biomedical Sciences, Korea University College of Medicine, Seoul, Republic of Korea; 5 Department of Biochemistry & Molecular Biology, Ajou University, School of Medicine, Suwon, Republic of Korea; 6 Department of Immunology, School of Medicine, Konkuk University, Chungju, Republic of Korea; 7 Center for Theragnosis, Biomedical Research Institute, Korea Institute of Science and Technology, Seoul, Republic of Korea; 8 Institute for Refractory Cancer Research, Samsung Medical Center, Seoul, Republic of Korea; 9 Samsung Advanced Institute for Health Sciences & Technology, Sungkyunkwan University School of Medicine, Seoul, Republic of Korea

**Keywords:** Targeted therapies, Ovarian cancer

## Abstract

Axitinib, small molecule tyrosine kinase inhibitor, demonstrates anti-cancer activity for various solid tumors. We investigated anti-cancer effect of axitinib in epithelial ovarian cancer (EOC). We treated EOC cells (A2780, HeyA8, RMG1, and HeyA8-MDR) with axitinib to evaluate its effects on cell viabilty, apoptosis and migration. Western blots were performed to assess VEGFR2, ERK, and AKT levels, and ELISA and FACS to evaluate apoptosis according to axitinib treatment. In addition, *in vivo* experiments in xenografts using A2780, RMG1, and HeyA8-MDR cell lines were performed. We repeated the experiment with patient-derived xenograft models (PDX) of EOC. Axitinib significantly inhibited cell survival and migration, and increased apoptosis in EOC cells. The expression of VEGFR2 and phosphorylation of AKT and ERK in A2780, RMG1, and HeyA8 were decreased with axitinib treatment in dose-dependent manner, but not in HeyA8-MDR. In *in vivo* experiments, axitinib significantly decreased tumor weight in xenograft models of drug-sensitive (A2780), and clear cell carcinoma (RMG1) and PDX models for platinum sensitive EOC compared to control, but was not effective in drug-resistant cell line (HeyA8-MDR) or heavily pretreated refractory PDX model. Axitinib showed significant anti-cancer effects in drug-sensitive or clear cell EOC cells via inhibition of VEGFR signals associated with cell proliferation, apoptosis and migration, but not in drug-resistant cells.

## Introduction

Epithelial ovarian cancer (EOC) is the most lethal gynecologic malignancy, and one of the leading causes of cancer-related death in women. The standard therapy for EOC consists of maximal surgical cytoreduction and adjuvant chemotherapy with taxane- and platinum-based chemotherapeutic agents. Despite previous investigations of novel chemotherapeutic regimens, and other targeted therapies, there was no significant improvements in clinical outcomes or cure rates, with current 5-year overall survival rates of 45%^1^.

Increased angiogenesis is related to progression of EOC, and a number of anti-angiogenic agents are under investigation as potential treatment options for advanced EOC. Currently, several target agents have reached phase 3 clinical trials for treatment of EOC^2^. Of these, bevacizumab (VEGF-A-specific humanized IgG1), an antiangiogenic agent, resulted in significant improvements of progression-free survival (PFS) when combined with chemotherapeutic agents, and has become a standard therapy for EOC in selected patients^3^. However, the overall survival benefit of bevacizumab seems insignificant considering its high medical expense. In addition, relapse after bevacizumab treatment suggests that there remains a need for alternative, potent, and multiple-target agents to counter tumor escape mechanisms.

Axitinib is a highly selective inhibitor of vascular endothelial growth factor receptor (VEGFR) tyrosine kinase 1, 2, and 3, and is reported to have the potential to control tumors and metastases by inhibiting angiogenesis and lymphangiogenesis, as well as via effects on tumor cells by apoptosis^4^. Clinical studies demonstrated promising anti-cancer activity in phase 2 trials for the treatment of various solid tumors. Axitinib showed single agent activity in patients with thyroid cancer^5^, nasopharyngeal cancer^6^, and resulted in improved response rate in recurrent glioblastoma patients^7^. Axitinib also significantly elongated PFS compared with sorafenib in patients with persistent renal cell carcinoma (RCC)^8^. Combination immunotherapy plus axitinib for the treatment of RCC resulted in encouraging antitumor activity^9^. However, the effects of axitinib in EOC have not been investigated.

The purpose of this study was to evaluate the anti-cancer effects of axinitib in EOC by using cell line xenografts and patient-derived xenograft (PDX) models, and to investigate the possible underlying mechanisms.

## Results

### Axitinib significantly affects cell viability and apoptosis of EOC cells

**Figure 1:**
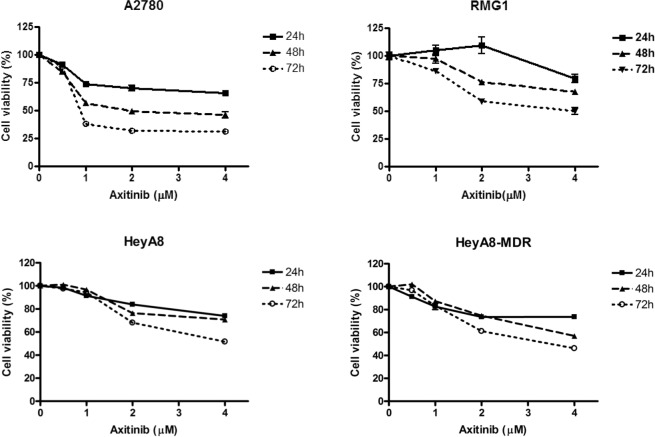
Cell viabilty. Reduced ovarian cancer cell viability following treatment with axitinib. Axitinib reduced cell viability in a dose-dependent manner, as evaluated by the MTT assay in A2780 (**A**), RMG1 (**B**), HeyA8 (**C**) and HeyA8-MDR (**D**) cells.

**Figure 2:**
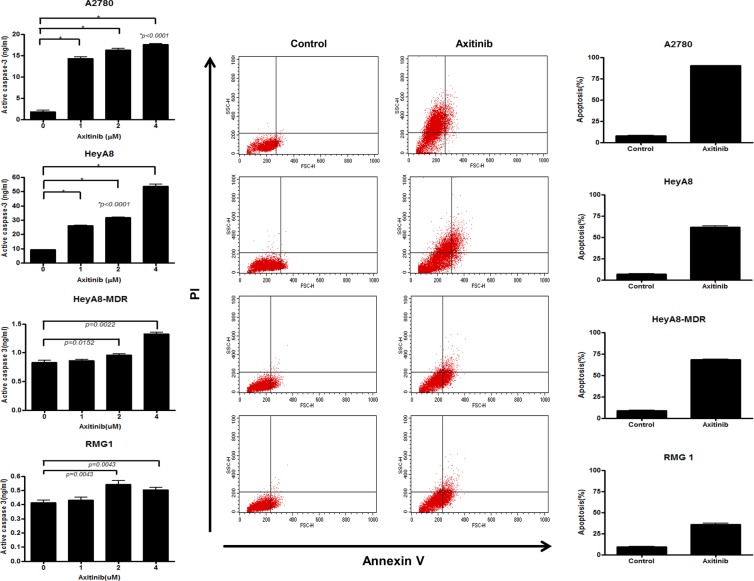
Apoptosis assay-active caspase 3 ELISA and FACS. Active caspase-3 ELISA (**A–D**) and flow cytometric determination (**E**) showed increased cell apoptosis in axitinib-treated cell lines. The significance of differences was determined by unpaired t-tests, and values of *P* < 0.05 (*) or *P* < 0.01 (**) were considered to be statistically significant.

Human EOC cells (A2780, RMG1, HeyA8, and HeyA8-MDR) were initially treated with axitinib for 24–72 h to confirm the inhibitory activity of axitinib on EOC viablity. Axitinib reduced viability of A2780, RMG1, HeyA8, and HeyA8-MDR EOC cells in a dose-dependent manner (Fig. 1). Axitinib had a time-dependent increasing inhibitory effect on cell viability at the same dose as the tested concentration. Apoptosis induction measured by active caspase-3 ELISA (24 h of treatment with 0, 1, 2, and 4 uM axitinib) and annexin V- FITC incorporation after treatment with axitinib (2 nM for A2780 and 4 nM for HeyA8, HeyA8-MDR, and RMG1) resulted in significantly increased apoptosis in axitinib-treated cells compared with control (Fig. 2, p < 0.0001 for A2780, HeyA8, p = 0.0022 for HeyA8-MDR, p = 0.0043 for RMG-1, respectively).

### Axitinib inhibits VEGFR2, AKT and ERK pathways in EOC cells

**Figure 3:**
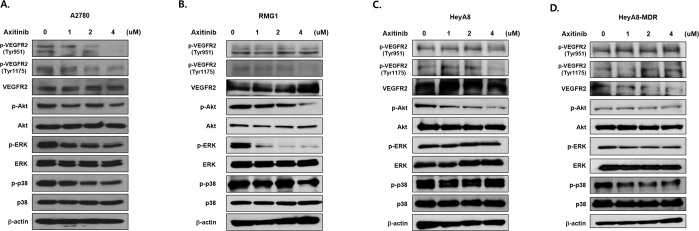
Cell signal analysis. VEGF2 signaling is down-regulated by axitinib. A2780 (**A**), RMG1 (**B**), HeyA8 (**C**), and HeyA8-MDR (**D**) cells were treated with various doses of axitinib, and VEGFR2 expression was determined by Western blot. Cells were treated for 4 h with the indicated doses of axitinib and analyzed by Western blot for activating the phosphorylation of VEGFR2 (Tyr-951/Tyr-1175), AKT, ERK, and p38. Phosphorylation of AKT, and ERK was inhibited after axitinib treatment in A2780, RMG1, and HeyA8, but did not change in HeyA8-MDR cells.

To evaluate the anti-cancer mechanism of axitinib in EOC cells, we assessed the VEGFR2, AKT and ERK pathways with Western blot. In experiments examining cell proliferation and apoptosis, axitinib was effective in A2780, RMG1, and HeyA8, and relatively ineffective in HeyA8-MDR. These cell lines were compared to determine the mechanism of action. Treatment with various doses of axitinib markedly decreased the expression of phospho-VEGFR2 in A2780, RMG1 and HeyA8 in a dose-dependent manner (Fig. 3A,B,C), but not in HeyA8-MDR cells (Fig. 3D). Phosphorylation of AKT, and ERK, a direct binding partner of VEGFR2, was also examined. Phosphorylation of AKT, and ERK was inhibited 4 h after axitinib treatment in A2780, RMG1 and HeyA8 cells, but this change was not observed in HeyA8-MDR or drug-resistant EOC cells.

### Axitinib inhibits cell migration in EOC cells

**Figure 4:**
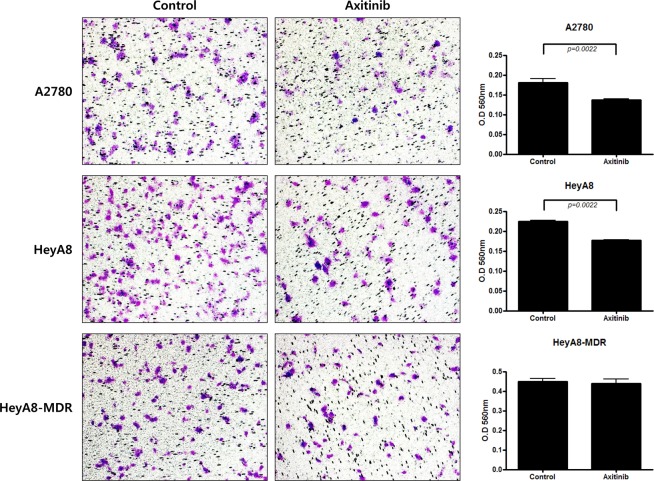
Migration assay. In axitinib-treated groups, the number of migrated EOC cells decreased per x200 fields (*P* = 0.0022).

Based on the results of a study of fetal lung adenocarcinoma showing that axitinib affects cell migration^10^, we performed cell migration assays in EOC cells. These assays revealed that axitinib-treated (24 h in 2 and 4 uM) EOC cells were less proficient at migrating than controls, with less absorbance observed at 560 nm (Fig. 4). In axitinib treatment groups, the number of migrated cells significantly decreased per x200 fields in A2780 (p = 0.0022) and HeyA8 (p = 0.0022), but not in HeyA8-MDR cells.

### Axitinib significantly inhibits tumor growth in cell line orthotopic xenografts of EOC

**Figure 5:**
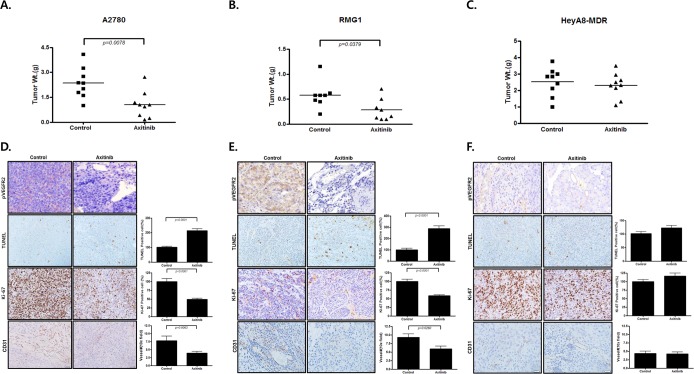
*In vivo* EOC cell line mouse models. Axitinib inhibits the tumor growth of ovarian cancer xenografts. Mice treated with axitinib had significantly lower tumor weight than control mice (by 50%; P < 0.005 in A2780 and RMG1), but the difference was not significant in drug-resistant EOC models (HeyA8-MDR). The expression of apoptosis,cell proliferation, and angiogenesis in xenografts was also analyzed by IHC with p-VEGFR2, TUNEL assay, Ki-67 staining, and CD31 staining.

To investigate the clinical relevance of our *in vitro* results, we conducted *in vivo* experiments using EOC orthotopic mouse models. A2780, RMG1, and HeyA8-MDR EOC cells were implanted into the peritoneal cavities of female nude mice, and therapy was started with axitinib (30 mg/kg twice daily p.o.) 7 days after cell injection. In A2780 and RMG1 models, the tumor weight of the axitinib-treated group had significantly decreased by 50% compared with controls (Fig. 5A,B, p = 0.0078, and p = 0.0379, respectively), but the difference was not significant in HeyA8-MDR models (Fig. 5C). Daily monitoring of animals throughout the therapy showed acceptable tolerability with no untoward side effects such as changes in body weight, mobility, posture, or feeding habits.

To validate the results of *in vitro* studies, we evaluated the effects of axitinib therapy on cell proliferation and apoptosis by immunohistochemistry for Ki-67 staining and TUNEL assays, respectively. Also, effects of axitinib on angiogenesis were evaluated by immunohistochemistry for CD31. The numbers of Ki-67 positive cancer cells were significantly lower in tumors from mice treated with axitinib than in tumors from controls in A2780 and RMG1 (Fig. 5D,E, p < 0.0001, and p < 0.0001, respectively), but not in a HeyA8-MDR mouse model (Fig. 5F). TUNEL assays showed that the number of apoptotic cancer cells was significantly higher in A2780 and RMG1 mouse models following therapy with axitinib. However, in the HeyA8-MDR cell line, differences between Ki 67 positive cells and TUNEL positive cells were insignificant. In the axitinib treated group, p-VEGFR2 positive cells were decreased in A2780 and RMG1 cell lines, but not in HeyA8-MDR. Number of vessels by CD31were significantly decreased in axitinib treated group of A2780 and RMG1 cell lines, but not in HeyA8-MDR.

### Axitinib inhibits tumor growth in EOC PDX models

We also examined the effects of axitinib in PDX models of EOCs using subrenal implantation of human EOC tissue. Our group previously developed PDX models of EOC^11^ We selected case numbers OV-89-M6, platinum-sensitive high grade serous carcinoma, OV-64-M9, clear cell carcinoma, and OV-40-M7, platinum-resistant recurrent high grade serous carcinoma. OV-89-M6 was a 53-year-old patient with FIGO stage IIIA2. She was treated with primary cytoreductive surgery followed by paclitaxel-carboplatin combination chemotherapy. There was no residual tumor after primary surgery, and her PFS was 28 months. OV-64-M9 was a 42-year-old patient with stage IIIC clear cell carcinoma with <1 cm residual disease after primary surgery. Progression of disease was detected during first-line chemotherapy consisting of paclitaxel-carboplatin, and the patient’s overall survival was only 2.4 months. OV-40-M7 was a 61-year-old patient with stage IV high grade serous carcinoma. The residual disease status after primary surgery was less than 1 cm, and the patient underwent 6 cycles of adjuvant paclitaxel-carboplatin combination chemotherapy. This case was classified as platinum resistant, as disease recurred after 6 months from end of first-line of chemotherapy.

**Figure 6:**
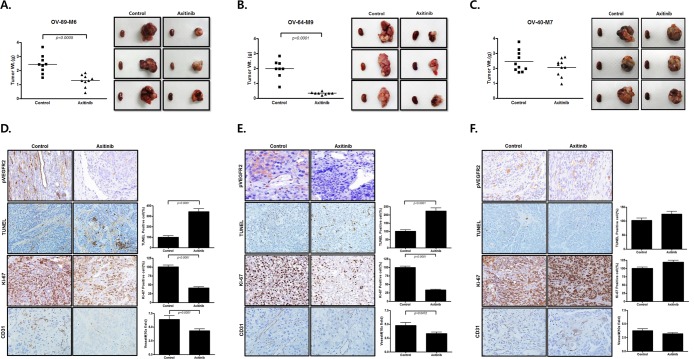
*In vivo* EOC PDX models. Axitinib inhibits tumor growth of ovarian cancer xenografts. Mice treated with axitinib had significantly lower tumor weight than control mice (*P* = 0.007 for OV-89-M5 and *P* < 0.0001 for OV-64-M9), but the effect was not significant in platinum-resistant OV-40-M7. The expression of apoptosis and cell proliferation in these xenografts was analyzed by IHC with p-VEGFR2(x400), Ki-67 staining, CD31 staining, and TUNEL assay (x200).

Treatment with axitinib significantly decreased tumor weight in two PDX models compared with the control group (*P* = 0.0005 for OV-89-M6 and *P* < 0.0001 for OV-64-M9, respectively) (Fig. 6A,B). The inhibitory effect of axitinib on tumor growth was not seen in the OV-40-M7 model, which is heavily-pretreated and platinum-resistant (Fig. 6C). Immunohistochemistry staining of Ki-67, p-VEGFR2, and TUNEL assay yielded similar results to those obtained for the xenograft model. Significantly higher numbers of TUNEL positive cells, and lower numbers of Ki-67 positive cells, were observed with axitinib treatment in high grade serous and clear cell carcinoma PDX (Fig. 6D,E, p < 0.0001). In platinum resistant ovarian cancer PDX, differences between controls and the axitinib-treated group for Ki-67 positive cells and apoptotic cells were not significant (Fig. 6F). In addition, in the axitinib-treated group, the number of p-VEGFR2 positive cells was lower in platinum sensitive high grade serous and clear cell carcinoma PDX, but this difference was not observed in platinum resistant high grade serous cases. Also, in the axitinib-treated group, the number of vessels by CD31 was decreased in platinum sensitive high grade serous and clear cell carcinoma PDX, but this difference was not observed in platinum resistant high grade serous cases.

## Discussion

Axitinib, a highly selective VEGFR tyrosine kinase inhibitor, is known as one of the most effective substance for the metastatic renal cell carcinoma treatment^12^. In this study, we exhibited anti-angiogenesis and anti-tumor activity of axitinib that has potential for use in the treatment of EOC. In an *in vitro* study, axitinib significantly inhibited proliferation and migration, and increased apoptosis, of EOC cells in a dose-dependent manner. Initially, cell viability experiments presented that axitinib showed cytotoxic activity in all EOC cells. In addition, axitinib-induced apoptosis was confirmed in EOC cell lines. However, in Western blot confirming expression of VEGFR and its downstream signaling in EOC cell lines, axitinib-induced inhibitory effects in VEGFR2, phosphorylation of AKT, and ERK were not observed in HeyA8-MDR. Unlike A2780 and HeyA8, the migration assay showed no effect of axitinib on HeyA8-MDR. Based on these results, we hypothesize that axitinib inhibits EOC cells by targeting multiple pathways including angiogenesis, AKT, and ERK signaling pathways. Additionally, invasion-related MMP2/ MMP9 ELISA was performed for further explanation of the differences between cell viability experiments and cell signal assay, but no differences were found in HeyA8-MDR and other cell lines. This may be due to differences in tumor microenvironment, but the exact mechanism through the experiment could not be presented. In orthotopic mouse models, tumor reduction by axitinib in platinum sensitive cell line and ovarian clear cell line was observed, but axitinib was not effective in tumor of drug resistant cell line. The same result was observed in the PDX model. Thus, in our experiments, we concluded that antitumor effects of axitinib as a single agent were significant only in drug-sensitive EOC models, but were not remarkable in drug-resistant EOC cell line xenograft or PDX models.

In our study, axitinib showed weaker effects in the platinum-resistant group than the platinum-sensitive group. Drug resistance can be explained by changes of intracellular active drug concentrations, drug-target interactions, target-mediated cell damage, damage-induced apoptotic signaling, or apoptotic effectors^13^, which may influence response to axitinib. In a previous study analyzing the biological characteristics of platinum-resistant cells^14^, resistant cell lines exhibited decreased levels of DNA platination and faster repair of damaged DNA, suggesting that drug uptake, detoxification, and excretion, along with the DNA repair pathway play central roles in resistant phenotypes. In the current study, axitinib was effective for treatment against a drug-resistant cell line (HeyA8-MDR) in cell viability assay and apoptosis assay, but not in cell signal assay and *in vivo* experiments with a drug-resistant xenograft model. We additionally retried Western blot to confirm expression of VEGFR and its downstream signaling for HeyA8 and HeyA8-MDR. Expression of phospho-VEGFR2, phosphorylation of AKT, and ERK was inhibited 4 h after axitinib treatment in HeyA8 cells, but in contrast, this change was not observed in HeyA8-MDR. To explain the difference between result of cell viability test and cell signal analysis, we performed invasion-related MMP2/9 ELISA in HeyA8, and HeyA8-MDR. However, result for MMP2 was not measured for HeyA8-MDR, and expression inhibition of MMP2 by axitinib showed no difference between HeyA8 and HeyA8-MDR as shown in Supplementary Data. Unfortunately, these additional experiments could not reveal a clear mechanism. These differences may be explained by differences in reactions between cell lines and tissue. In cancer research, *in vitro* experiments are mainly performed to study gene regulation and signaling that lead to uncontrolled cell growth. *In vivo* experiments are performed to evaluate cancer cell interactions with the environment, and result in more informative outcomes because the microenvironment is a critical determinant of the migration strategy and the efficiency of cancer cell invasion^15^.

VEGF-mediated angiogenesis plays an important role in ovarian function, and there is a well-investigated association between VEGF overexpression, increased angiogenesis, and the development and progression of ovarian cancer^16^. Previously in clinical studies, high serum VEGF levels were correlated with higher risks of recurrence and death of EOC in a review of nine studies including 529 EOC patients^17^. Serum VEGF was considered an independent prognostic factor for survival after multivariate analysis in five studies. Associations between increased angiogenesis and progression of EOC led to the investigation of a number of anti-angiogenic agents as potential treatment options for EOC. Bevacizumab gained approval for first-line treatment for advanced EOC patients, and is included in the National Comprehensive Cancer Network (NCCN) guidelines for EOC treatment. Other anti-angiogenic agents, including trebananib, aflibercept, nintedanib, cediranib, imatinib, pazopanib, sorafenib and sunitinib, are currently in phase II/III development^18^. However, the effects of axitinib, part of a new generation of tyrosine kinase agents, differ from those of previously existing agents due to its greater activity and potency of inhibition of VEGFR1-3^19^. These attributes had not previously been investigated in EOC.

Strategies for inhibiting angiogenesis are key to prevent the survival, proliferation, invasion, and metastasis of ovarian cancer cells^20^. In this study, we found that axitinib is effective as a single agent in a drug-sensitive EOC cell line mouse and PDX models, but not in drug-resistant EOC cell models. In clinical trials using anti-angiogenic agents for the treatment of EOC, the response rate was not significant in drug-resistant recurrent EOC. A study of other VEGFR inhibitor, sorafenib, showed no anti-tumor activity in patients with possibly drug resistant EOC or primary peritoneal carcinoma after multiple use of chemotherapy^21^. Previous study of bevacizumab as a single agent in patients with platinum-resistant relapsed EOC and peritoneal serous carcinoma also showed poor response (response rate 15.9% (7/44) and median response duration 4.2 months (range, 1.7 to 9.2 months))^22^.

To the best of our knowledge, this is the first assessment of the efficacy and mechanism of axitinib as an anti-cancer therapeutic in preclinical models of EOC. In this study, we demonstrated marked anti-tumor effects of axitinib that were associated with anti-angiogenesis in drug-sensitive EOC cells, xenograft, and PDX models. Our findings have important clinical implications for the administration of axitinib as a single agent in the treatment of drug-sensitive EOC patients who are highly likely to experience toxicity if treated with conventional taxane- and platinum-based chemotherapy. However, the effects of axitinib were not promising against drug-resistant EOC, so clinical trials evaluating combination therapies of axitinib with other target agents, including immunotherapy, are needed. In summary, axitinib may be one of the most promising VEGFR tyrosine kinase inhibitors available, exhibiting significant antitumor activity when used as a single agent for the treatment of EOC.

## Materials and Methods

### Chemicals and cell culture

Axitinib (AG 013736) was acquired from APExBIO Tech LLC (Houston, TX, USA). A2780 and RMG1 were acquired from the European Collection of Authenticated Cell Cultures (ECACC, Salisbury, SP4 0JG, UK) and the Health Science Research Resources Bank (JCRB, Osaka, Japan). HeyA8 and HeyA8-MDR were gifted from Dr. Anil K. Sood (Department of Cancer Biology, M.D. Anderson Cancer Center, University of Texas, USA). A2780, HeyA8 and HeyA8-MDR were kept in RPMI 1640 supplemented with 10% fetal bovine serum (FBS). RMG1 was kept in Ham’s F12 supplement with 10% FBS. All cells were kept in 5% CO_2_ at 37 °C.

### Cell viability assay

Cells were plated in culture medium in 96-well plates at 3 × 10^3^ cells/well. After 24 h, cells were treated with axitinib, and assays performed at 24, 48, and 72 h. For cell viability assays, cells were stained with 3-(4, 5-dimethylthiazol-2-yl)-2,5-diphenyltetrazolium bromide (MTT; Amresco, Solon, OH, USA); after 4 h of additional incubation, the medium was discarded, 100ul of acidic isopropanol (0.1 N HCL in absolute isopropanol) was added, and the plate was shaken gently. Absorbance was measured on an enzyme linked immunosorbent assay (ELISA) reader at a wavelength of 540 nm. Experiment was conducted as our previous study^23^.

### Active caspase-3 ELISA

For the apoptosis assay, we used an active caspase-3 ELISA assay (#KHO1091; Invitrogen). Cells were seeded in 6-well plates (1 × 10^4^ cells in 3 ml of media per well), and incubated overnight to allow the cells to attach to the plate. After 24 h of treatment with 0, 1, 2, and 4 uM axitinib, the medium was removed by suction and cells were lysed with lysis buffer. Apoptotic activity was determined for each well according to the manufacturer’s protocol, as described previously^24^.

### FACS analysis

Cell apoptosis was measured at 48 h after treatment using the FITC Annexin-V apoptosis Detection Kit-1 (BD Pharmingen, San Diego, CA, USA) according to the manufacturer’s protocol. Each sample was assayed in triplicate. A minimum of 5,000 cells were then analyzed by FACScan with Cell Quest software (Beckton Dickinson) for acquisition and analysis, as described previously^23^.

### Western blot

Cells were lysed in PRO-PRE-Protein Extraction Solution (Intron Biotechnology, Seongnam, Korea). Protein concentrations were determined using a Bradford assay kit (BIO-RAD, Hercules, CA, USA). Cell lysates (50 *μ*g of total protein) were separated in 8% acrylamide gels by sodium dodecyl sulfate-polyacrylamide gel electrophoresis (SDS-PAGE) and transferred to Hybond-ECL nitrocellulose filter paper (Amersham Biosciences, Buckinghamshire, UK). Membranes were blocked with 5% BSA in Tris-buffered saline containing 0.1% Tween-20 for 1 h at room temperature. Protein bands were probed with VEGFR2 antibody (Santa Cruz Biotechnology, Santa Cruz, CA, USA) at a 1:500 dilution; phospho-VEGFR2 (Tyr951, Tyr1175), total-ERK (Thr202/Tyr204), phosphor-ERK (Thr202/Tyr204), total-AKT, phospho-AKT total-p38, phospho-p38 (Cell Signaling, USA) at 1:1000 dilutions; *β*-actin antibody at a 1:4000 dilution (Santa Cruz Biotechnology, Santa Cruz, USA) and then labeled with horseradish peroxidase-conjugated anti-rabbit antibody (GE Healthcare, Piscataway, USA). Bands were visualized by enhanced chemiluminescence using an ECL kit (Amersham Biosciences, Buckinghamshire, UK) according to the manufacturer’s protocol, as described previously^23^.

### Migration assay

The migration assay was performed with a Cytoselect 24-well cell migration kit according to the manufacturer’s protocol (Cell Biolabs, San Diego, USA), as described previously^25^.

### Animal care and development of *in vivo* models including established cell lines and PDX

*In vivo* experiments were performed to confirm the anti-tumor effect of axitinib in orthotopic cell-lines or patient-derived xenograft (PDX) mouse models. Female BALB/c nude mice were purchased from ORIENT BIO (Sungnam, Korea). This study was performed in accordance with all relevant guidelines and regulations. This study was reviewed and approved by the Institutional Animal Care and Use Committee (IACUC) of Samsung Biomedical Research Institute (SBRI). SBRI is an Association for Assessment and Accreditation of Laboratory Animal Care International-accredited facility (AAALAC International, protocol No. H-A9-003) and abides by the Institute of Laboratory Animal Resources (ILAR) guidelines (IRB no. 2015-08-046).

To generate tumors, A2780 (1.0 × 10^6^ cells/0.2 mL HBSS), RMG1 (5.0 × 10^6^ cells/0.2 mL HBSS), and HeyA8-MDR (2.5 × 10^5^ cells/0.2 mL HBSS) were injected into the peritoneal cavities of BALB/c nude mice that were 6 to 8 weeks old^23^. To generate PDX models of EOC, three tumor specimens retrieved from human patients during surgery were cut into small pieces (less than 2–3 mm in diameter), implanted into the subrenal capsule of the left kidney in mice^11^, and propagated by serial transplantation. Tumors were derived from one patient each with platinum-sensitive high grade serous EOC (OV-89-M5), clear cell carcinoma (OV-64-M9), and shigh grade serous EOC, platinum-resistant recurrent (OV-40-M7).

After 7 days of cell injection for the cell line models or 5 weeks for the PDX models, mice (n = 10 per group) were subjected to experimental treatments. The control group was given 0.5% methyl cellulose and the axitinib group was given 30 mg/kg axitinib twice daily orally. Mice were monitored daily for tumor development and postoperative complications, and were sacrificed on days 35 to 40 or if they seemed moribund. Total body weight and tumor weight of each mouse were recorded. Tumors were fixed in formalin and embedded in paraffin or snap frozen in OCT compound (Sakura Finetek Japan, Tokyo, Japan) in liquid nitrogen^23^.

### Immunohistochemical analysis

The primary antibodies used had action against p-VEGFR2 (Abcam, ab38464). Tissue sections were deparaffinized three times in xylene for a total of 15 mins and subsequently rehydrated. Immunostaining for p-VEGFR2 was performed using a Bond-maxTM automated immunostainer (Leica Biosystems, Melbourne, Australia) and the BondTM Polymer Refines Detection kit (Vision Biosystems, Melbourne, Australia). Briefly, antigen retrieval was carried out at 97 °C for 20 mins in ER1 buffer. After blocking endogenous peroxidase activity with 3% hydrogen peroxidase for 10 mins, primary antibody incubation was carried out for 15 mins at room temperature at an antibody dilution of 1:200. Negative controls (with substitution of TBS for primary antibody) were performed simultaneously. Immunohistochemical staining for Ki-67 (NOVUS, NB 600–1252), and CD31 (ABCAM, AB28364) was performed as described previously^26^. Apoptotic positive cells were analyzed by TUNEL assay using the ApopTag Peroxidase *in situ* Apoptosis kit (Millipore, S7100) as described previously^27^.

### Data analysis

The Mann–Whitney U test was used to evaluate the significance of differences among groups for both *in vitro* and *in vivo* assays. All statistical tests were two-sided, and *p* values less than 0.05 were considered to be significant. SPSS software (version 17.0; SPSS, Chicago, IL, USA) was used for statistical analyses.

## Supplementary information

Supplementary information.

Supplementary dataset.

## Data Availability

No datasets were generated or analysed during the current study.

## References

[1] Huang L, Cronin KA, Johnson KA, Mariotto AB, Feuer EJ (2008). Improved survival time: what can survival cure models tell us about population-based survival improvements in late-stage colorectal, ovarian, and testicular cancer?. Cancer.

[2] Bax HJ (2016). Therapeutic targets and new directions for antibodies developed for ovarian cancer. MAbs.

[3] Burger RA (2011). Incorporation of bevacizumab in the primary treatment of ovarian cancer. The New England journal of medicine.

[4] Hu-Lowe DD (2008). Nonclinical antiangiogenesis and antitumor activities of axitinib (AG-013736), an oral, potent, and selective inhibitor of vascular endothelial growth factor receptor tyrosine kinases 1, 2, 3. Clinical cancer research: an official journal of the American Association for Cancer Research.

[5] Molina-Vega M (2018). Tyrosine kinase inhibitors in iodine-refractory differentiated thyroid cancer: experience in clinical practice. Endocrine.

[6] Hui EP (2018). Efficacy, Safety, and Pharmacokinetics of Axitinib in Nasopharyngeal Carcinoma: A Preclinical and Phase II Correlative Study. Clinical cancer research: an official journal of the American Association for Cancer Research.

[7] Duerinck J (2018). Randomized phase II trial comparing axitinib with the combination of axitinib and lomustine in patients with recurrent glioblastoma. Journal of neuro-oncology.

[8] Rini BI (2011). Comparative effectiveness of axitinib versus sorafenib in advanced renal cell carcinoma (AXIS): a randomised phase 3 trial. Lancet (London, England).

[9] Atkins MB (2018). Axitinib in combination with pembrolizumab in patients with advanced renal cell cancer: a non-randomised, open-label, dose-finding, and dose-expansion phase 1b trial. The Lancet. Oncology.

[10] Menna C (2014). Axitinib affects cell viability and migration of a primary foetal lung adenocarcinoma culture. Cancer investigation.

[11] Heo EJ (2017). Patient-Derived Xenograft Models of Epithelial Ovarian Cancer for Preclinical. Studies. Cancer research and treatment: official journal of Korean Cancer Association.

[12] Akaza H, Fukuyama T (2014). Axitinib for the treatment of advanced renal cell carcinoma. Expert opinion on pharmacotherapy.

[13] Agarwal R, Kaye SB (2003). Ovarian cancer: strategies for overcoming resistance to chemotherapy. Nature reviews. Cancer.

[14] Sonego M (2017). Common biological phenotypes characterize the acquisition of platinum-resistance in epithelial ovarian cancer cells. Scientific reports.

[15] Brabek J, Mierke CT, Rosel D, Vesely P, Fabry B (2010). The role of the tissue microenvironment in the regulation of cancer cell motility and invasion. Cell communication and signaling: CCS.

[16] Masoumi Moghaddam S, Amini A, Morris DL, Pourgholami MH (2012). Significance of vascular endothelial growth factor in growth and peritoneal dissemination of ovarian cancer. Cancer metastasis reviews.

[17] Bandiera E (2012). Prognostic significance of vascular endothelial growth factor serum determination in women with ovarian cancer. ISRN obstetrics and gynecology.

[18] Aravantinos G, Pectasides D (2014). Bevacizumab in combination with chemotherapy for the treatment of advanced ovarian cancer: a systematic review. Journal of ovarian research.

[19] Gross-Goupil M, Francois L, Quivy A, Ravaud A (2013). Axitinib: a review of its safety and efficacy in the treatment of adults with advanced renal cell carcinoma. Clinical Medicine Insights. Oncology.

[20] Hefler LA (2006). Preoperative serum vascular endothelial growth factor as a prognostic parameter in ovarian cancer. Gynecologic oncology.

[21] Bodnar L, Górnas M, Szczylik C (2011). Sorafenib as a third line therapy in patients with epithelial ovarian cancer or primary peritoneal cancer: A phase II study. Gynecologic oncology.

[22] Cannistra SA (2007). Phase II study of bevacizumab in patients with platinum-resistant ovarian cancer or peritoneal serous cancer. Journal of clinical oncology: official journal of the American Society of Clinical Oncology.

[23] Choi CH (2017). The anti-cancer effects of itraconazole in epithelial ovarian cancer. Scientific reports.

[24] Song T (2012). Expression of 67-kDa laminin receptor was associated with tumor progression and poor prognosis in epithelial ovarian cancer. Gynecol Oncol.

[25] Majid S (2013). miRNA-34b inhibits prostate cancer through demethylation, active chromatin modifications, and AKT pathways. Clin Cancer Res.

[26] Zillhardt M, Christensen JG, Lengyel E (2010). An orally available small-molecule inhibitor of c-Met, PF-2341066, reduces tumor burden and metastasis in a preclinical model of ovarian cancer metastasis. Neoplasia (New York, N.Y.).

[27] Inoue K (2000). Paclitaxel enhances the effects of the anti-epidermal growth factor receptor monoclonal antibody ImClone C225 in mice with metastatic human bladder transitional cell carcinoma. Clinical cancer research: an official journal of the American Association for Cancer Research.

