# Supplementary material for: Preclinical assessment of the VEGFR inhibitor axitinib as a therapeutic agent for epithelial ovarian cancer
Source: Sci Rep. 2020 Mar 17;10:4904. doi: 10.1038/s41598-020-61871-w (PMC7078214; doi:10.1038/s41598-020-61871-w)
Supplement: Supplementary file 1 — Supplementary information. [file 41598_2020_61871_MOESM1_ESM.pdf]

# Preclinical assessment of the VEGFR inhibitor axitinib as a therapeutic agent for epithelial ovarian cancer

E Sun Paik[1,]*, Tae-Hyun Kim[2,]*, Young Jae Cho[3,]*, Jiyoon Ryu[3], Jung-Joo Choi[3], Yoo-Young Lee[3], Tae-Joong Kim[3], Chel-Hun Choi[3], Woo Young Kim[1], Jason K. Sa[4], Jin-Ku Lee[5], Byoung-Gie Kim[3], Duk-Soo Bae[3], Hee Dong Han[6], Hyung Jun Ahn[7], Jeong-Won Lee[3,8,9,] **

[1]Department of Obstetrics and Gynecology, Kangbuk Samsung Hospital, Sungkyunkwan University School of Medicine, Seoul, Republic of Korea

[2]Department of Obstetrics and Gynecology, Konyang University Hospital, Daejon, Republic of Korea

[3]Department of Obstetrics and Gynecology, Samsung Medical Center, Sungkyunkwan University School of Medicine, Seoul, Republic of Korea

[4]Department of Biomedical Sciences, Korea University College of Medicine, Seoul, Republic of Korea

[5]Department of Biochemistry & Molecular Biology, Ajou University, School of Medicine, Suwon, Republic of Korea

[6]Department of Immunology, School of Medicine, Konkuk University, Chungju, Republic of Korea

[7]Center for Theragnosis, Biomedical Research Institute, Korea Institute of Science and Technology, Seoul, Republic of Korea

[8]Institute for Refractory Cancer Research, Samsung Medical Center, Seoul, Republic of Korea.

[9]Samsung Advanced Institute for Health Sciences & Technology, Sungkyunkwan University School of Medicine, Seoul, Republic of Korea

*These authors contributed equally to this work.

**Corresponding Author:

Jeong-Won Lee, MD, PhD

Department of Obstetrics and Gynecology, Samsung Medical Center, Sungkyunkwan University School of Medicine, 81 Irwon-ro, Gangnam-gu, Seoul 06351, Korea. Tel.: +82-2-3410-1382; Fax: +82-2-3410-0630. E-mail address: garden.lee@samsung.com (J.-W. Lee)

**Supplementary file 1.** Invasion assay-MMP2/MMP9 ELISA in HeyA8 and HeyA8-MDR.

**Supplementary file 2.** Full-length gels and blots for figure 3C (HeyA8).

**Supplementary file 3.** Full-length gels and blots for figure 3D (HeyA8-MDR).
