# Supplementary material for: Preclinical assessment of the VEGFR inhibitor axitinib as a therapeutic agent for epithelial ovarian cancer
Source: Sci Rep. 2020 Mar 17;10:4904. doi: 10.1038/s41598-020-61871-w (PMC7078214; doi:10.1038/s41598-020-61871-w)

**Supplementary file 1.** Invasion assay-MMP2/MMP9 ELISA in HeyA8 and HeyA8-MDR.


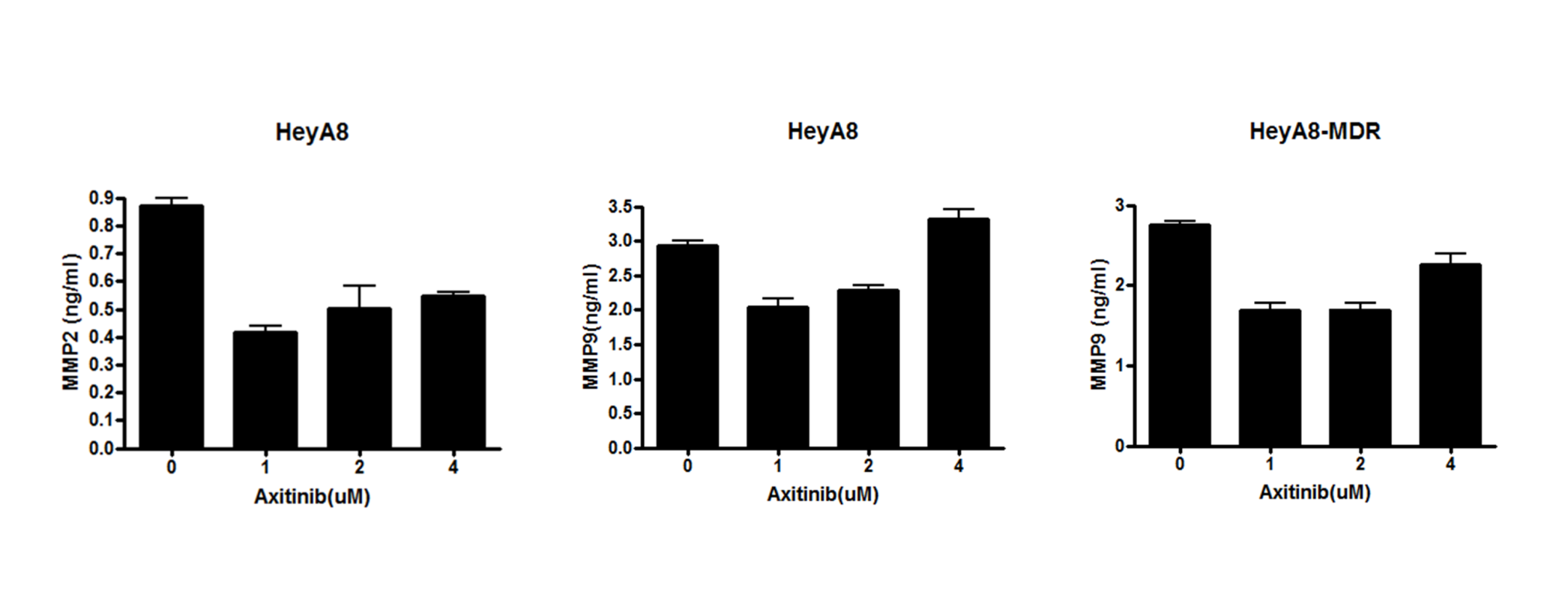


**Supplementary file 2.** Full-length gels and blots for figure 3C (HeyA8).


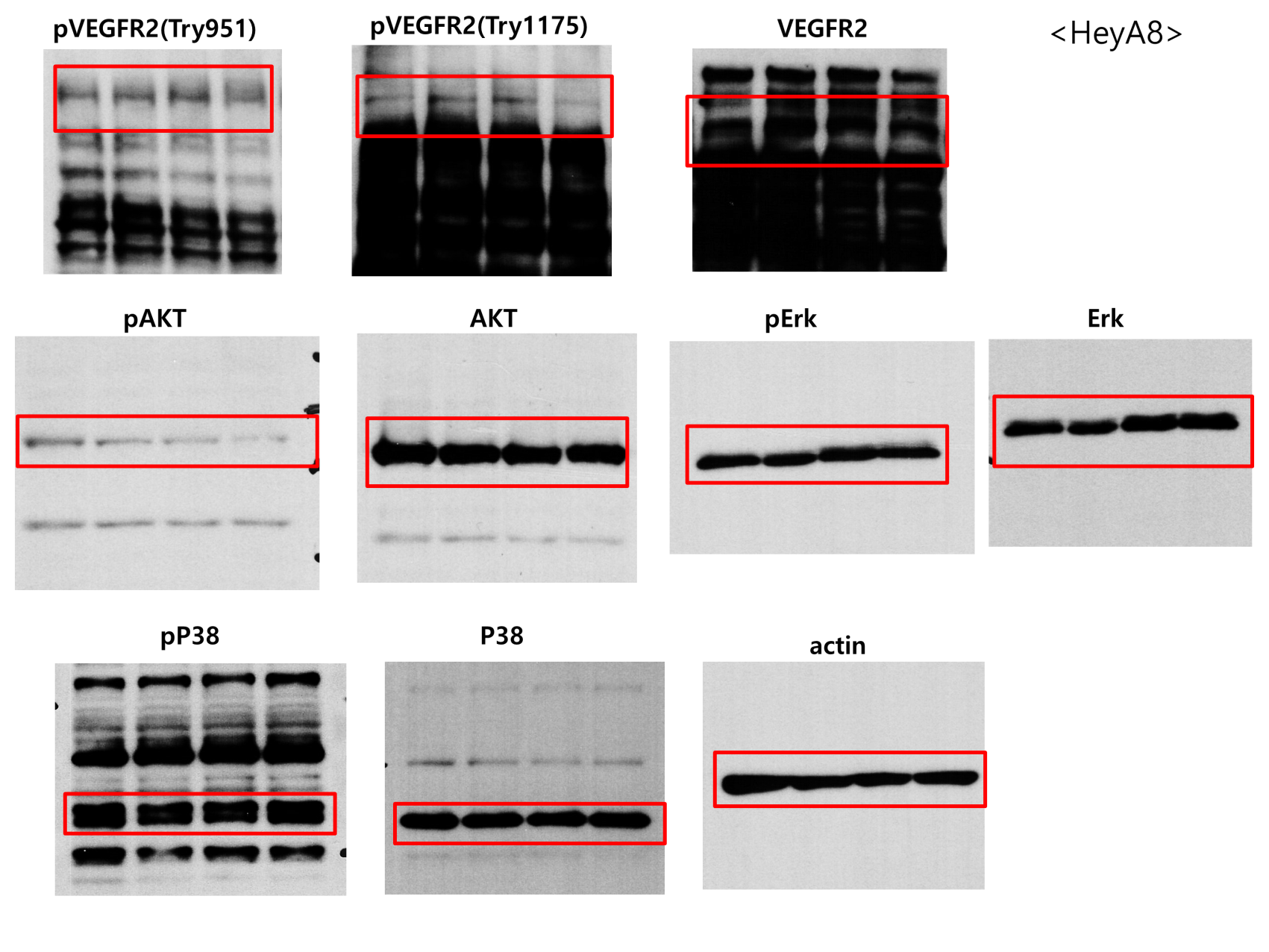


**Supplementary file 3.** Full-length gels and blots for figure 3D (HeyA8-MDR).


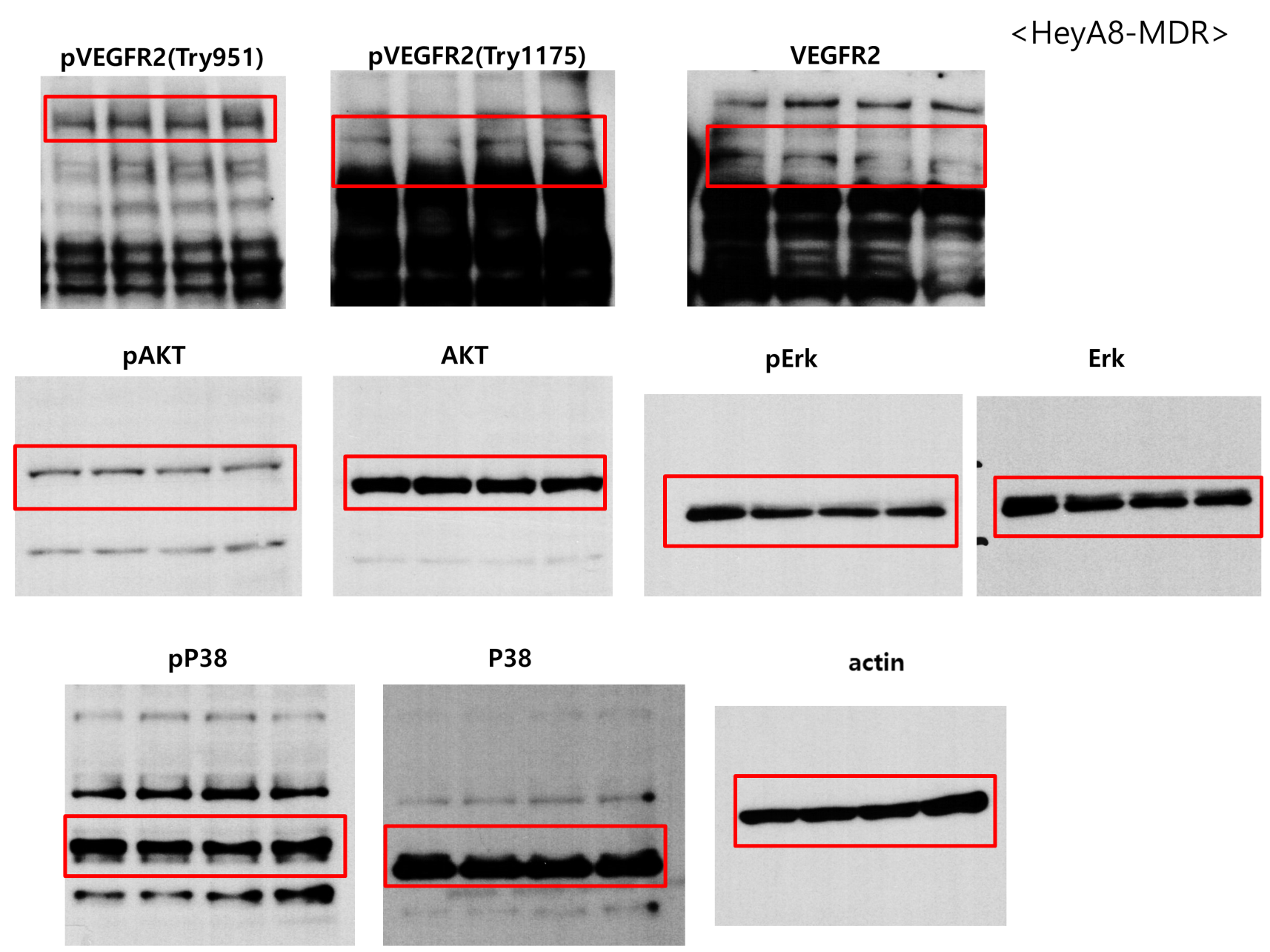

Supplement: Supplementary file 2 — Supplementary dataset. [file 41598_2020_61871_MOESM2_ESM.docx]
